# Lipofection-Mediated Introduction of CRISPR/Cas9 System into Porcine Oocytes and Embryos

**DOI:** 10.3390/ani11020578

**Published:** 2021-02-23

**Authors:** Maki Hirata, Manita Wittayarat, Zhao Namula, Quynh Anh Le, Qingyi Lin, Koki Takebayashi, Chommanart Thongkittidilok, Fuminori Tanihara, Takeshige Otoi

**Affiliations:** 1 Faculty of Bioscience and Bioindustry, Tokushima University, Tokushima 770-8501, Japan; mhirata@tokushima-u.ac.jp (M.H.); zhaonamula@gdou.edu.cn (Z.N.); leanhquynh@gmail.com (Q.A.L.); 13794341393@163.com (Q.L.); tkb.315.koki@gmail.com (K.T.); otoi@tokushima-u.ac.jp (T.O.); 2 Faculty of Veterinary Science, Prince of Songkla University, Songkhla 90110, Thailand; mwittayarat@gmail.com; 3 College of Coastal Agricultural Sciences, Guangdong Ocean University, Zhanjiang 524088, China; 4 Akkhraratchakumari Veterinary College, Walailak University, Nakorn Sri Thammarat 80161, Thailand; thongkittidilokc@gmail.com; 5 Center for Development of Advanced Medical Technology, Jichi Medical University, Tochigi 329-0498, Japan

**Keywords:** CRISPR/Cas9, embryo, lipofection, oocyte, pig

## Abstract

**Simple Summary:**

Liposome-mediated gene transfer has become an alternative method for establishing a gene targeting framework, and the production of mutant animals may be feasible even in laboratories without specialized equipment. However, whether blastocyst genome editing can be performed by treatment with lipofection reagent, guide RNA, and Cas9, without performing electroporation or microinjection, remains unclear. In this study, we demonstrated that lipofection treatment successfully induced mutation into zygotes during in vitro fertilization and in embryos at the 2- and 4-cell stages. Although liposome-mediated gene editing is a feasible system for use with zona-pellucida-free oocytes/embryos, several challenges must be overcome.

**Abstract:**

Liposome-mediated gene transfer has become an alternative method for establishing a gene targeting framework, and the production of mutant animals may be feasible even in laboratories without specialized equipment. However, how this system functions in mammalian oocytes and embryos remains unclear. The present study was conducted to clarify whether blastocyst genome editing can be performed by treatment with lipofection reagent, guide RNA, and Cas9 for 5 h without using electroporation or microinjection. A mosaic mutation was observed in blastocysts derived from zona pellucida (ZP)-free oocytes following lipofection treatment, regardless of the target genes. When lipofection treatment was performed after in vitro fertilization (IVF), no significant differences in the mutation rates or mutation efficiency were found between blastocysts derived from embryos treated at 24 and 29 h from the start of IVF. Only blastocysts from embryos exposed to lipofection treatment at 29 h after IVF contained biallelic mutant. Furthermore, there were no significant differences in the mutation rates or mutation efficiency between blastocysts derived from embryos at the 2- and 4-cell stages. This suggests that lipofection-mediated gene editing can be performed in ZP-free oocytes and ZP-free embryos; however, other factors affecting the system efficiency should be further investigated.

## 1. Introduction

Clustered regularly interspaced short palindromic repeats (CRISPR) and CRISPR-associated protein 9 (Cas9) have been developed and widely employed for the rapid generation of genetically modified pigs [1,2]. To generate these pigs, modern techniques such as somatic cell nuclear transfer, microinjection, and electroporation have been used [2,3,4]. However, these methods require costly equipment such as micromanipulators or electroporators, which are required to introduce the CRISPR/Cas9 system into oocytes/zygotes. If equipment-free methods can be established for pig zygotes produced by in vitro maturation and in vitro fertilization (IVF) techniques, the rapid and large-scale production of mutant pigs may be feasible even in unequipped laboratories.

Lipofection, defined as liposome-mediated transfection, involves introducing foreign genes into mammalian cells using lipophilic reagents that enhance the cellular uptake of polynucleotides [5]. Lipofection was first developed by Felgner et al. [6] by using a cationic liposomeDNA complex to deliver DNA plasmid across the plasma membrane through a fusion process involving endosomes. Since then, this technique has been used in several types of cells, including primary endothelial cells [7], secretory epithelial cells [8], endometrial stromal cells [9], primary culture of astrocytes [10,11], and oligodendrocytes [12].

DNA constructs may also be transfected into numerous embryonic cell types via lipofection [13,14,15]. However, the efficacy of lipofection-mediated gene editing in oocytes and embryos is unclear, including in experimental animals. Lipofection treatment shows potential for delivering gene-editing-associated proteins into cells [16]. The present study was conducted to determine whether lipofection of the CRISPR/Cas9 system, Cas9 protein, and guide RNA (gRNA) into zona pellucida (ZP)-free oocytes and ZP-free embryos can be used for gene editing in the resulting blastocysts without using equipment for microinjection and electroporation. Our results indicate that lipofection-mediated gene editing can be applied to introduce the CRISPR/Cas9 system into ZP-free oocytes and ZP-free embryos.

## 2. Materials and Methods

### 2.1. Ethical Approval

Animal experiments were approved by the Institutional Animal Care and Use Committee of Tokushima University (approval number: T2019-11).

### 2.2. Oocyte Collection and In Vitro Maturation

Pig ovaries were obtained from prepubertal gilts (Landrace × Large White × Duroc breeds) at a local slaughterhouse and transported at 30 °C in physiological saline within 1 h to the laboratory. The ovaries were washed three times with prewarmed physiological saline solution supplemented with 100 IU/mL penicillin G potassium (Meiji, Tokyo, Japan) and 0.1 mg/mL streptomycin sulfate (Meiji). Follicles with diameters of 3–6 mm on the ovarian surface were sliced on a sterilized dish using a surgical blade, and cumulus-oocyte complexes (COCs) were visualized and collected under a stereomicroscope. Approximately 50 COCs were cultured in 500 µL maturation medium consisting of tissue culture medium 199 with Earle’s salts (Thermo Fisher Scientific, Waltham, MA, USA) supplemented with 10% (*v*/*v*) porcine follicular fluid, 0.6 mM cysteine (Sigma-Aldrich, St. Louis, MO, USA), 50 µM β-mercaptoethanol (Wako Pure Chemical Industries Ltd., Osaka, Japan), 50 µM sodium pyruvate (Sigma-Aldrich), 2 mg/mL D-sorbitol (Wako Pure Chemical Industries Ltd.), 10 IU/mL equine chorionic gonadotropin (Kyoritu Seiyaku, Tokyo, Japan), 10 IU/mL human chorionic gonadotropin (Kyoritu Seiyaku), and 50 µg/mL gentamicin (Sigma-Aldrich), and then covered with mineral oil (Sigma-Aldrich) for 22 h in 4-well dishes (Nunc A/S, Roskilde, Denmark). The COCs were transferred into maturation medium without hormones for an additional 22 h of culture. The COCs were incubated at 39 °C in a humidified incubator containing 5% CO_2_.

### 2.3. IVF and Embryo Culture

The matured oocytes were subjected to IVF as described previously [17]. Briefly, frozen-thawed ejaculated spermatozoa were transferred into 5 mL of fertilization medium (PFM; Research Institute for the Functional Peptides Co., Yamagata, Japan) and washed by centrifugation at 500× *g* for 5 min. The pelleted spermatozoa were resuspended in fertilization medium and adjusted to 1 × 10^6^ cells/mL. Approximately 50 oocytes were transferred into 500 µL of sperm-containing fertilization medium, covered with mineral oil in 4-well dishes, and co-incubated for 5 h at 39 °C in a humidified incubator containing 5% CO_2_, 5% O_2_, and 90% N_2_. After co-incubation, the attached spermatozoa were gently removed from the oocytes by mechanical pipetting. The putative zygotes were transferred to porcine zygote medium (PZM-5; Research Institute for the Functional Peptides Co.) and cultured for 3 days. The embryos were then cultured in porcine blastocyst medium (PBM; Research Institute for the Functional Peptides Co.) for 4 days to evaluate their ability to develop to the blastocyst stage and the genotype of the resulting blastocysts.

### 2.4. Lipofection-Mediated Introduction of CRISPR/Cas9 System

The ZP of matured oocytes and embryos at the 1-cell to 8-cell stage was removed prior to lipofection-mediated gene editing. Oocytes and embryos were exposed to 0.5% (*w*/*v*) actinase-E (Kaken-Seiyaku Corp.) in Dulbecco’s PBS (Thermo Fisher Scientific) for 20–30 s, transferred to PFM and PZM-5, respectively, and then freed completely from their ZP by gentle pipetting. ZP-free oocytes and embryos were incubated at 39 °C in a humidified incubator containing 5% CO_2_, 5% O_2_, and 90% N_2_ for 1 h before the lipofection-mediated introduction of the CRISPR/Cas9 system using lipofectamine 2000 (LF2000, Thermo Fisher Scientific).

Lipofection solution was prepared by diluting 5 µL of LF2000 with 20 μL Opti-MEM I solution (Thermo Fisher Scientific) and then mixing with 25 μL of Nuclease-Free Duplex Buffer (IDT, Integrated DNA Technologies, Coralville, IA, USA) containing 200 ng/μL gRNA (Alt-R CRISPR crRNAs and tracrRNA, chemically modified and length-optimized variants of native guide RNAs purchased from IDT) and 600 ng/μL Cas9 protein (Guide-it Recombinant Cas9, Takara Bio, Shiga, Japan). Lipofection solution (50 μL) was added to 450 μL PFM or PZM-5 and used for lipofection-mediated gene editing by 5 h of co-incubation with ZP-free oocytes/embryos in a humidified incubator containing 5% CO_2_, 5% O_2_, and 90% N_2_. As a control for analyzing embryonic development, some ZP-free oocytes and embryos without lipofection treatment were cultured in the same manner.

### 2.5. Analysis of the Targeted Gene in Embryos

Genomic DNA was isolated from the blastocysts by boiling in 50 mM NaOH solution. After neutralization, the DNA samples were subjected to polymerase chain reaction (PCR) using KOD One PCR Master Mix (Toyobo, Osaka, Japan) according to the manufacturer’s instructions and using the following specific primers: 5′-ATAGAAGTCCAAATATTTTCCCCGC-3′ (forward) and 5′-ACCTCGTACGGGGAGATGTC-3′ (reverse) for *PDX1*, and 5′-AGTCAGGATGCTTCCCCTTT-3′ (forward) and 5′-AAGCTGGTGACTTGGCTGAT-3′ (reverse) for *GGTA1*. The PCR products were extracted by agarose gel electrophoresis using a Fast Gene Gel/PCR Extraction Kit (Nippon Genetics, Tokyo, Japan). The targeted genomic regions of the PCR products were directly sequenced by Sanger sequencing using the BigDye Terminator Cycle Sequencing Kit version 3.1 (Thermo Fisher Scientific) and an ABI 3500 genetic analyzer (Applied Biosystems, Foster City, CA, USA). The TIDE (tracking of indels by decomposition) bioinformatics package was used to determine the genotype of each blastocyst [18]. Blastocysts were classified as biallelic mutation (carrying no wild-type (WT) sequences), mosaics (carrying more than one type of mutation and the WT sequence), or WT (carrying only the WT sequence). The editing rate was defined as the ratio of the number of gene-edited blastocysts to the total number of sequenced blastocysts. Editing efficiency was defined as the proportion of indel mutation events in blastocysts carrying the mosaic or biallelic mutation products.

### 2.6. Experimental Design

We evaluated the development of ZP-free mature oocytes and embryos subjected to lipofection-mediated gene editing, as well as the efficiency of gene editing in the resulting blastocysts.

#### 2.6.1. Experiment 1: Lipofection-Mediated Introduction of CRISPR/Cas9 System into Oocytes during IVF

gRNAs targeting *PDX1* (5′-TGGCGAGGAGCAGTACTACG-3′), which have been confirmed to show high genome editing efficiency [19], and gRNA targeting *GGTA1* (5′- TGTTTTGGGAATACATCAAC-3′), which was used to produce *GGTA1*-deficient pigs [20], were used to evaluate the gene editing of porcine oocytes by lipofection during IVF. Matured oocytes were denuded from the cumulus cells by mechanical pipetting with 0.1% (*w*/*v*) hyaluronidase; subsequently, the ZP of matured oocytes was removed prior to IVF. Lipofection solution (50 μL) was added to 450 μL PFM containing ZP-free mature oocytes and spermatozoa, and they were co-incubated for 5 h during IVF. After 5 h of incubation, ZP-free putative zygotes were cultured for 7 days as described above.

#### 2.6.2. Experiment 2: Lipofection-Mediated Introduction of CRISPR/Cas9 System into Embryos

To improve the gene-editing efficiency, we focused on the gRNA exhibiting lower mutation rates in Experiment 1. To evaluate the effects of the post-IVF exposure period of lipofection treatment on embryonic development and editing efficiency, embryos collected at 24 and 29 h from the start of IVF were freed from the ZP. Lipofection solution (50 μL) was added to 450 μL PZM-5 containing ZP-free embryos, co-incubated for 5 h, and then cultured for 6 days as described above.

#### 2.6.3. Experiment 3: Comparison of Lipofection-Mediated Gene-Editing Efficiency among Embryos at Different Cleavage Stages

To evaluate the effects of embryonic stage on editing efficiency, embryos at the 1- to 8-cell stage were collected at 29 h from the start of IVF and freed from the ZP. Lipofection solution (50 μL) was added to 450 μL PZM-5 containing ZP-free embryos, co-incubated for 5 h, and then cultured for 6 days as described above.

### 2.7. Statistical Analyses

The percentages of embryos that cleaved and developed to the blastocyst stage were subjected to arcsine transformation before analysis of variance (ANOVA). The transformed data were evaluated using ANOVA, followed by the protected Fisher’s least significant difference tests. The analysis was performed using StatView software (Abacus Concepts, Berkeley, CA, USA). Data were checked for normality and homoscedasticity prior to the use of ANOVA (Shapiro–Wilk’s test and Levene’s test). The percentages of mutated blastocysts were analyzed using chi-squared tests with Yates’ correction. The percentages of mutation efficiency were evaluated using an independent Student’s *t*-test. Differences with a probability value (*p*) of 0.05 or less were considered as statistically significant.

## 3. Results

### 3.1. Experiment 1

**Figure 1:**
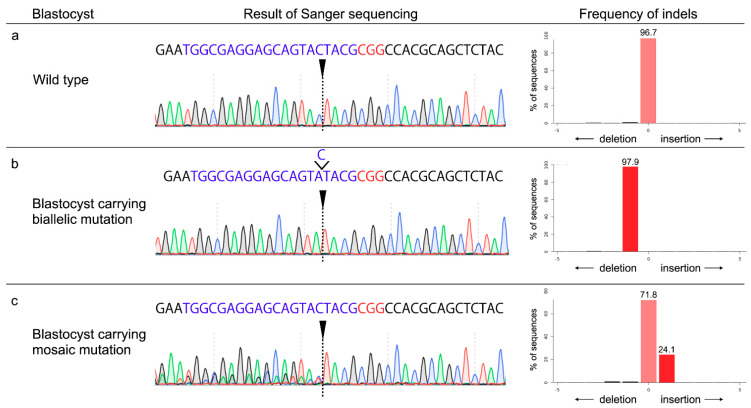
Representative results of the Sanger sequencing of blastocysts formed after lipofection-mediated gene editing with guide RNA (gRNA) targeting *PDX1* and frequencies of indels decomposed from Sanger sequence data by tracking of indels by decomposition (TIDE) analysis. (**a**) Analysis of wild-type blastocyst. (**b**) Analysis of a blastocyst carrying biallelic mutation. The Sanger sequence trace consists of a 1-bp deleted sequence trace without the wild-type sequence. TIDE analysis compared the Sanger sequence data with wild-type sequence data and shows 1 bp deleted. (**c**) Analysis of blastocyst carrying mosaic mutation. The Sanger sequence traces of a blastocyst carrying mosaic mutation consist of composite sequence traces after the break site. TIDE analysis decomposed the Sanger sequence data, and wild-type and indel mutation with their frequencies are indicated. Arrowheads indicate the predicted Cas9 cleavage sites. Nucleotides in blue color indicate target sequences, nucleotides in red color indicate protospacer adjacent motif (PAM) sequences, and nucleotides in green indicate the deleted nucleotide.

When the developmental competence of ZP-free mature oocytes subjected to lipofection-mediated gene editing during IVF was examined, there were no significant differences in the blastocyst formation rates between the gRNAs targeting *PDX1* and *GGTA1* (Table 1). Moreover, the blastocyst formation rates of ZP-free oocytes after LF2000 treatment were similar to those of control ZP-free oocytes. Figure 1 shows representative results of the Sanger sequencing of blastocysts formed after lipofection-mediated gene editing with gRNA and the frequencies of indels decomposed from Sanger sequence data by TIDE analysis. The blastocysts from ZP-free oocytes treated with LF2000 contained mosaic mutants but not biallelic mutants, irrespective of the target genes. There were no significant differences in the mutation rates or mutation efficiency of the resulting blastocysts between gRNAs targeting *PDX1* and *GGTA1*.

### 3.2. Experiment 2

The results of Experiment 1 showed that there were no significant differences in the mutation rates or mutation efficiency of the resulting blastocysts between gRNAs targeting *PDX1* and *GGTA1*. However, the mutation rate induced by gRNA targeting *PDX1* was lower than that by gRNA targeting *GGTA1*. To improve the lower mutation rate, we used gRNA targeting *PDX1* in Experiments 2 and 3.

When the effects of the post-IVF exposure period of LF2000 treatment on embryonic development were examined using gRNA targeting *PDX1*, the blastocyst formation rates of ZP-free embryos treated with LF2000 were significantly lower (*p* < 0.05) than those of ZP-free control embryos, irrespective of the post-IVF exposure period (Table 2). When the target sequence of the resulting blastocysts was assessed, there were no significant differences in the mutation rates or mutation efficiency between blastocysts derived from embryos treated at 24 and 29 h from the start of IVF. However, the biallelic mutation was detected only in blastocysts from embryos exposed to LF2000 treatment at 29 h from the start of IVF.

### 3.3. Experiment 3

**Figure 2:**
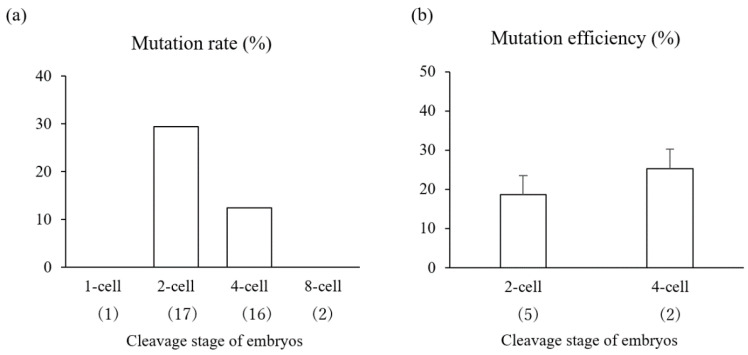
Mutation rate (**a**) and mutation efficiency (**b**) of blastocysts derived from zona pellucida (ZP)-free embryos at different cleavage stages subjected to lipofection-mediated gene editing with gRNA targeting *PDX1*. Embryos at the 1- to 8-cell stage were collected at 29 h from the start of in vitro fertilization and then exposed to LF2000, gRNA, and Cas9 protein for 5 h. (**a**) Mutation rate was defined as the ratio of the number of edited blastocysts to the total number of sequenced blastocysts. (**b**) Mutation efficiencies represent the proportion of indel mutation events in gene-edited blastocysts. The numbers within parentheses indicate the total number of blastocysts examined. Four replicate trials were carried out. Each bar represents the mean ± SEM.

When the effects of embryonic stage on editing efficiency were evaluated, only mosaic mutations were found in blastocysts derived from embryos at the 2- and 4-cell stages. However, there were no significant differences in the mutation rates (Figure 2a) or mutation efficiency (Figure 2b) between blastocysts from embryos at the 2- and 4-cell stages (29.4% vs. 12.5% and 18.6% vs. 25.3%, respectively).

## 4. Discussion

Successful gene delivery by liposome-mediated transfection is rate-limited by numerous biological barriers, including cellular uptake, intracellular trafficking, endosomal escape, and nuclear entry [21,22,23]. As lipofectamine, a type of cationic liposome, has shown the potential to increase the final transfection efficiency across a broad range of cell types, it is recognized as the gold standard for safely delivering exogenous genes into targeted cells [22]. In the present study, we investigated whether the lipofection of a ribonucleoprotein (RNP), consisting of the Cas9 protein in complex with a targeting gRNA, into ZP-free oocytes and ZP-free embryos without using electroporation and microinjection can produce gene-edited blastocysts.

In Experiment 1, in the presence of 5 µL LF2000 in a transfection volume of 500 µL for 5 h, the blastocyst formation rates of transfected ZP-free oocytes and control ZP-free oocytes were similar. This suggests that the concentration of LF2000 used in this study was virtually nontoxic to porcine oocytes, likely because of the large size of oocytes. These oocytes have a low surface-to-volume ratio, making it difficult for LF2000 to move across the cell plasma membranes, resulting in low toxicity [24,25]. However, all mutated blastocysts derived from ZP-free oocytes treated with LF2000 were genetically mosaic upon the use of gRNAs targeting either *PDX1* or *GGTA1*, whose gene editing efficiency was confirmed in our previous study [19,20]. These results indicate that the timing of RNP introduction into oocytes may not be proper for producing biallelic mutant blastocysts. Previous studies demonstrated that the direct injection of the CRISPR/Cas9 system into porcine zygotes at 6 h after IVF or 8 h after parthenote embryo activation achieved high biallelic mutations and low mosaicism [2,26,27]. This may be because CRISPR/Cas9 component mRNAs were actively used for protein synthesis in oocytes after injection, which was likely associated with pronucleus-formation-triggered mRNA translation, as the ribosomes involved in protein synthesis are covered at the outer nuclear membrane [26]. In this context, determining the time at which oocyte transfection with RNP occurs may be helpful for overcoming such mosaicism.

In Experiment 2, we tested this hypothesis by assessing the effects of the post-IVF exposure period of LF2000 treatment on embryonic development and mutation rates using gRNA targeting *PDX1*. The mutation rates and mutation efficiency in this experiment were clearly improved compared to those in which oocyte transfection was performed pre-IVF. Our results appear to validate previous findings showing that mRNA translation is likely to increase in oocytes with distinct nuclear membrane (pronucleus formation), resulting in a greater mutation efficiency [26]. In this study, we delivered Cas9 protein/gRNA complexes, which require no mRNA translation. In mice, major zygotic genome activation with an open chromatin state occurs during the extended G2 phase in 2-cell stage embryos [28,29]. In contrast, major genome activation has been reported to occur at the 4-cell stage in porcine embryos [30,31]. The open chromatin state may improve the accessibility of the CRISPR system to the target site, and the duration of the target residence of CRISPR/Cas9 correlates with cleavage activity [32]. These studies support our results of increased gene editing events in post-IVF embryos. Furthermore, in Experiment 3, when lipofection-mediated gene editing was introduced into 2- and 4-cell embryos, the genome editing efficiency was similar. Although the only biallelic mutant (7.1%) detected in this experiment was from blastocysts derived from embryos transfected with LF2000 at 29 h after the start of IVF, most blastocysts remained mosaic, regardless of the exposure period from IVF. If Cas9 protein/gRNA complexes remain active throughout the cell division process, mosaicism can occur as early as the 2-cell stage [26,33,34,35]. Morphologic evaluation in porcine embryos produced in vitro was reported by Wang et al. [36], who found that 25% of cleaved embryos were first observed at 24 h after IVF, before significantly increasing to 71% at 30 h after IVF. This suggests that the transfection timing of the CRISPR/Cas9 system at 24 and 29 h after IVF were slightly late for generating biallelic mutations, as cell division may have already occurred after 24 h. Moreover, because 93% of fertilized porcine oocytes had formed both male and female pronuclei at 12 h after IVF [36], performing lipofection slightly earlier than 12 h after IVF is a key point for resolving mosaicism.

Lipofection-based transfection exhibits distinct mobility phases. The liposome first binds to the cell surface and slowly moves across the cell plasma membrane into the cytosol. Next, LF2000 accompanies actin filaments, leading to anomalous and confined diffusion before being transported along with the microtubules, resulting in perinuclear accumulation [22]. This reveals that actin filaments are critical for the intracellular trafficking of lipofection-based transfection reagents. Our results in Experiment 3 showed that although the low mutation efficiency and resulting low number of gene-edited blastocysts induced statistical variability, the mutation rates of blastocysts were gradually decreased with increasing embryonic stage. According to Wang et al. [36], 31% of 2-cell and 18% of 4-cell stage porcine embryos showed actin filaments in the perinuclear area of all blastomeres, whereas most 8-cell stage embryos only had partial perinuclear actin filaments in the blastomeres. If the number of actin filaments is small, the lipofection-based transfection system may not function well, explaining why blastocysts derived from embryos at the 8-cell stage tended to yield a poorer rate of mutation compared to embryos at the 2- and 4-cell stages. However, at 24 h to 29 h from the start of IVF, the normal fertilized embryos are at the 2–4-cell stage, indicating that embryos at the 8-cell stage underwent cytoplasm fragmentation and that some 1-cell stage embryos had already lost their developmental ability. This is one of the limitations of our study, and further investigation of the timing of lipofection treatment is required.

In our lipofection-mediated system, gene-editing efficiency was insufficient compared with electroporation and microinjection-mediated gene editing [20,37], resulting in a low number of biallelic mutant blastocysts. Our previous study demonstrated that the results of electroporation- and microinjection-mediated gene editing were also affected by the concentration of CRISPR/Cas9 components, voltage strength during electroporation, and the developmental stage of zygotes/embryos [37,38,39]. Further optimization is crucial for the practical application of the lipofection-mediated gene editing system during embryogenesis.

## 5. Conclusions

We demonstrated that lipofection-mediated gene editing without electroporation or microinjection is a feasible system for introducing the Cas9 protein and gRNA into ZP-free oocytes and ZP-free embryos, particularly when using embryos at the 2- and 4-cell stages by treatment with LF2000 for 5 h to produce gene-edited blastocysts. However, other factors also affect the system efficiency, such as the time point at which the oocytes/zygotes/embryos are transfected. These factors should be further investigated to improve the use of this gene delivery system.

## Figures and Tables

**Table 1:** Development of zona pellucida (ZP)-free embryos subjected to lipofection-mediated gene editing with gRNA targeting *PDX1* or *GGTA1* during in vitro fertilization (IVF) ^†^.

gRNA ^††^	No. of Embryos Examined	No. (Mean ± SEM) of Embryos Developed to Blastocysts	No. of Blastocysts Examined	No. (Mean) of Blastocysts ^†††^			Mutation Efficiency (Mean ± SEM) ^††††^
				WT	Biallelic	Mosaic	
Control	202	24 (11.9 ± 0.4)	-	-	-	-	-
*PDX1*	395	44 (9.5 ± 2.6)	35	32 (91.4)	0 (0)	3 (8.6)	35.8 ±10.5
GGTA1	272	39 (13.8 ± 6.0)	39	33 (84.6)	0 (0)	6 (15.4)	17.8 ± 3.0

^†^ Five replicate trials were carried out. Percentages are expressed as the mean ± SEM; ^††^ ZP-free mature oocytes were exposed to LF2000, gRNA, and Cas9 protein for 5 h with spermatozoa, and cultured for 7 days; ^†††^ Proportions were calculated by dividing the number of blastocysts by the total number of examined blastocysts; ^††††^ Mean proportions represent the proportion of indel mutation events in mosaic blastocysts determined by the TIDE bioinformatics package. WT: wild-type. Biallelic: biallelic mutation. Mosaic: mosaic mutation.

**Table 2:** Development of zona pellucida (ZP)-free embryos subjected to lipofection-mediated gene editing with gRNA targeting *PDX1* after IVF ^†^.

Post-IVF Exposure Period ^††^	No. of Embryos Examined	No. (Mean ± SEM) of Embryos Developed to Blastocysts	No. of Blastocysts Examined	No. (Mean) of Blastocysts ^†††^			Mutation Efficiency (Mean ± SEM) ^††††^
				WT	Biallelic	Mosaic	
Control	210	45 (21.4 ± 2.2) ^a^	-	-	-	-	-
24–29 h	250	21 (8.1 ± 1.5) ^b^	18	8 (44.4)	0 (0)	10 (55.6)	45.3 ± 9.9
29–34 h	250	20 (8.1 ± 0.9) ^b^	14	6 (42.9)	1 (7.1)	7 (50.0)	40.8 ± 10.1

^†^ Four replicate trials were carried out. Percentages are expressed as the mean ± SEM; ^††^ ZP-free embryos were collected at 24 and 29 h from the start of in vitro fertilization, and then exposed to LF2000, gRNA, and Cas9 protein for 5 h, and cultured for 6 days. As a control, ZP-free embryos without LF2000 treatment were cultured for the same number of days; ^†††^ Proportions were calculated by dividing the number of blastocysts by the total number of examined blastocysts; ^††††^ Mean proportions represent the proportion of indel mutation events in mosaic and biallelic blastocysts determined by the TIDE bioinformatics package; WT: wild-type; Biallelic: biallelic mutation; Mosaic: mosaic mutation; ^a,b^ Values with different superscripts in the same column are significantly different (*p* < 0.05).

## Data Availability

The data presented in this study are available on request from the corresponding author. The data are not publicly available to preserve privacy of the data.
